# Alterations in Prefrontal-Limbic Functional Activation and Connectivity in Chronic Stress-Induced Visceral Hyperalgesia

**DOI:** 10.1371/journal.pone.0059138

**Published:** 2013-03-19

**Authors:** Zhuo Wang, Marco A. Ocampo, Raina D. Pang, Mihail Bota, Sylvie Bradesi, Emeran A. Mayer, Daniel P. Holschneider

**Affiliations:** 1 Department of Psychiatry and Behavioral Sciences, University of Southern California, Los Angeles, California, United States of America; 2 Program in Neuroscience, University of Southern California, Los Angeles, California, United States of America; 3 Department of Biological Sciences, University of Southern California, Los Angeles, California, United States of America; 4 Departments of Neurology, Biomedical Engineering, Cell & Neurobiology, University of Southern California, Los Angeles, California, United States of America; 5 Veterans Administration Greater Los Angeles Healthcare System, Los Angeles, California, United States of America; 6 Center for Neurobiology of Stress, Department of Medicine, University of California Los Angeles, Los Angeles, California, United States of America; 7 Departments of Physiology, Psychiatry and Biobehavioral Sciences, University of California Los Angeles, Los Angeles, California, United States of America; Centre national de la recherche scientifique, France

## Abstract

Repeated water avoidance stress (WAS) induces sustained visceral hyperalgesia (VH) in rats measured as enhanced visceromotor response to colorectal distension (CRD). This model incorporates two characteristic features of human irritable bowel syndrome (IBS), VH and a prominent role of stress in the onset and exacerbation of IBS symptoms. Little is known regarding central mechanisms underlying the stress-induced VH. Here, we applied an autoradiographic perfusion method to map regional and network-level neural correlates of VH. Adult male rats were exposed to WAS or sham treatment for 1 hour/day for 10 days. The visceromotor response was measured before and after the treatment. Cerebral blood flow (CBF) mapping was performed by intravenous injection of radiotracer ([^14^C]-iodoantipyrine) while the rat was receiving a 60-mmHg CRD or no distension. Regional CBF-related tissue radioactivity was quantified in autoradiographic images of brain slices and analyzed in 3-dimensionally reconstructed brains with statistical parametric mapping. Compared to sham rats, stressed rats showed VH in association with greater CRD-evoked activation in the insular cortex, amygdala, and hypothalamus, but reduced activation in the prelimbic area (PrL) of prefrontal cortex. We constrained results of seed correlation analysis by known structural connectivity of the PrL to generate structurally linked functional connectivity (SLFC) of the PrL. Dramatic differences in the SLFC of PrL were noted between stressed and sham rats under distension. In particular, sham rats showed negative correlation between the PrL and amygdala, which was absent in stressed rats. The altered pattern of functional brain activation is in general agreement with that observed in IBS patients in human brain imaging studies, providing further support for the face and construct validity of the WAS model for IBS. The absence of prefrontal cortex-amygdala anticorrelation in stressed rats is consistent with the notion that impaired corticolimbic modulation acts as a central mechanism underlying stress-induced VH.

## Introduction

Considerable evidence links stress with the onset and symptom exacerbation in irritable bowel syndrome (IBS) [1]–[3]. To better understand the underlying mechanisms underlying this stress sensitivity, and to identify novel targets for drug development, stress-based animal models for IBS have been established and extensively studied, using as stressors electric foot shock [4], maternal separation [5], social defeat and overcrowding [6], as well as repeated water avoidance stress (WAS) [7]. Visceromotor responses measured as abdominal electromyographic signals evoked by colorectal distension (CRD), are most commonly used to assess stress-induced visceral hyperalgesia, modeling a cardinal symptom of IBS. However, given the multidimensional nature of pain, the visceromotor response in rodents likely reflects only a portion of the complex human visceral pain experience. In recent years, functional brain imaging technology has emerged as a powerful tool to bridge the measurement gap between preclinical and clinical pain research, providing an objective measurement of pain in humans and laboratory animals alike. Comparing alterations in CRD-evoked brain responses in stress-induced visceral hyperalgesic rodents and that reported in IBS patients by human brain imaging studies can provide important validation for the stress-based animal models for human IBS. A better understanding of such stress-induced alterations in brain nociceptive responses is critical to delineating the underlying mechanisms.

There are few published reports on functional brain mapping studies in stress-induced visceral hyperalgesia animal models. Stam et al. [4] examined in rats the effect of foot shock on CRD-evoked expression of c-Fos, a gene marker of neuronal activity. In the central amygdala, as well as prelimbic, infralimbic, insular, and cingulate cortices, previously shocked rats showed *reduced* c-Fos expression following CRD compared with no-shock controls. Wouters et al. [8] used H_2_^15^O microPET to map CRD-evoked functional brain activation in maternal-separated rats before and 1 day after 1 hour of WAS. Following WAS, rats showed CRD-evoked activation in new areas, including the somatosensory cortex and hippocampus, and greater deactivation in the frontal cortex. While these studies provided important evidence that stress-induced visceral hyperalgesia is associated with alterations in brain responses to CRD, due to the use of anesthesia in both studies, it is difficult to compare the results directly with human brain imaging findings [9].

We have recently adapted an autoradiographic cerebral blood flow (CBF) perfusion mapping method to the rat CRD model [10]. In contrast to the requirement of sedation or restraint in fMRI and microPET studies, the perfusion method allows functional brain mapping in awake and nonrestrained rats. This is particularly important when studying brain mechanisms related to stress and affect related pain modulation, as brain networks involved in nociception, stress and affect significantly overlap, and are subject to influences by anesthetics [9], [11]. Using this method, we have shown that patterns of brain activation in response to acute CRD and in expectation of CRD in the rat are in general agreement with that reported in the human brain imaging literature [10], [12], [13]. Here, we applied perfusion mapping to characterize the effect of repeated WAS, which we have previously shown to induce long-lasting visceral hyperalgesia [7], on CRD-evoked functional brain activation.

Repeated, daily WAS (7–10 days) induces a chronic visceral hyperalgesia in the rodent model. This hyperalgesia persists for periods as long as one month after cessation of the stress [7], [14], something not seen after single day stress exposure [15]. These observations suggest that chronic/subchronic stress results in a functional reorganization of the nociceptive response. It has been proposed that an important brain mechanism underlying stress-induced visceral hyperalgesia involves chronic stress induced impairment of prefrontal-cortico-limbic pain modulation [16]. The prefrontal cortex (PFC) has been implicated in this corticolimbic regulatory circuit. To examine this hypothesis, we applied seed-region correlational analysis to assess changes in CRD-evoked functional connectivity (FC) of the PFC in the rat following stress. The prelimbic cortex (PrL) of the PFC was chosen as the seed based on our previously reported findings of robust activation of this region both during acute CRD [10] and in expectation of CRD [13], and the observation that activity of PrL and amygdala were anticorrelated (e.g. showed a negative correlation) during expectation of CRD [13].

One major limitation of FC analysis is that correlation based analysis does not address causality. Furthermore, due to multiple comparisons, false positive findings are inevitable when a simple significance threshold is applied. Constraining FC analysis with structural connectivity (SC) information can reduce the number of false positive reports, as well as provide directionality for the otherwise non-directional FC networks. The concept of anatomically constrained FC analysis has been implemented in effective connectivity analysis of human and animal brain imaging data [17], [18], but has been largely limited to small-scale networks. Recent studies have suggested a direct association between FC and SC in the human brain by combining resting-state fMRI with structural diffusion tensor imaging (DTI) measurement [19]. With the recent surge in efforts to construct connectome databases for human and rodent brain, it has become possible to combine and compare SC and FC at the whole brain level. Here, we constrained FC analysis with *complete* SC information of the PrL based on reports from tract tracing experiments manually collated in the Brain Architecture Management System (BAMS, http://brancusi.usc.edu/) [20], [21]. The resulting *structurally linked functional connectivity* (SL-) network keeps only functional connections over direct structural projections. An SLFC network inherits directionality information from the SC network and the sign of functional interaction (positive or negative) from the FC network, constituting a substantive step toward understanding the causality in brain circuits.

## Materials and Methods

### Animals

Adult male Wistar rats (2–2.5 month old) were purchased from Harlan Sprague Dawley (Indianapolis, IN, USA) and were individually housed in the vivarium on a 12-hour light/12-hour dark cycle with free access to water and rodent chow. All experiments were conducted under a protocol approved by the Institutional Animal Care and Use Committee of the University of Southern California, an institution accredited by the Association for Assessment and Accreditation of Laboratory Animal Care, International. All work was in accordance with the guidelines of the Committee for Research and Ethical Issues of the International Association or the Study of Pain. The numbers of animals in each group were as follows: Sham stress/0-mmHg CRD, n = 9; Sham stress/60-mmHg CRD, n = 10; WAS/0-mmHg CRD, n = 10; WAS/60-mmHg CRD, n = 10.

### Surgical procedures

**Figure 1:**
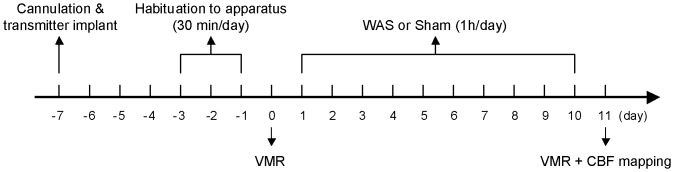
Experimental design. Visceral motor response (VMR) to colorectal distension (CRD) was measured before (day 0, baseline) and after (day 11) water avoidance stress (WAS) or sham treatment. Each time, CRDs of 10-, 20-, 40-, and 60-mmHg (duration = 20 s, interstimulus interval = 4 min) were delivered twice for each pressure level. On day 11, following VMR measurement, cerebral blood flow (CBF) mapping was performed while the animal was receiving a 60-mmHg CRD or no distension (0-mmHg control).

One week before the start of WAS treatment (Fig. 1), animals were anesthetized (isoflurane 2% in 70% oxygen and 30% nitrous oxide). The right external jugular vein was cannulated with a 5 French silastic catheter (Dow Corning Corp., Midland, MI, USA), advanced into the superior vena cava. The port at the distal end of the catheter was tunneled subcutaneously and externalized dorsally in the region rostral to the scapula. Subsequently, a telemetry transmitter was implanted to measure abdominal EMG. Such implants can be turned on and off with an external magnet and send a radiofrequency signal of EMG acitvity to a receiver platform placed underneath the rat's cage. The body of the transmitter (TA11-CTA-F40, Data Sciences Intl., St. Paul, MN, USA) was implanted subcutaneously on the dorsum of the animal caudal to the scapula. A skin incision was made on the abdomen and electrodes of the transmitter were tunneled subutaneously to the abdominal incision. Tips of the eletrodes were bared, placed in parallel (0.5 cm apart), and stitched into the left external oblique musculature, just superior to the inguinal ligament. The receiver platform was linked via a data exchange matrix to a PC computer. All animals were allowed to recover for seven days. The catheter was flushed every other day postoperatively to ensure patency (0.3 mL of sterile 0.9% saline, followed by 0.1 mL taurolidine-citrate catheter lock solution, Access Technologies, Skokie, IL, USA).

### Assessment and quantification of the VMR to CRD

VMR to CRD was assessed as described before [7]. Briefly, under light isoflurance anesthesia (1.5% isoflurance × 3 min), a flexible latex balloon (length = 6 cm) was inserted intra-anally such that its caudal end was 1 cm proximal to the anus. The silicon tubing connecting the balloon and the barostat (Distender Series II, G&J Eletronics Inc., Toronto, Canada) was fixed to the base of the tail with adhesive tape and covered by a stainless steel spring for protection against animal biting. Animals were allowed to recover for 30 min in the experiment cage, the floor of which was covered with bedding from the animal's home cage. The CRD procedure consisted of two series of phasic distension to constant pressure of 10, 20, 40, and 60 mmHg with 20-s duration and 4-min interstimulus intervals. The visceromotor response was quantified by measuring EMG activity in the external oblique musculature. EMG signals were recorded telemetrically at a sampling rate of 1 kHz, digitized and stored on a PC computer with the Dataquest ART 3.0 software (Data Sciences Intl., St. Paul, MN, USA). EMG waveforms were lowcut filtered at 20 Hz to eliminate movement interference, and then full-wave rectified. Area under the curve (AUC) was calculated for the 20-s distension period normalized by the 20-s before-distension baseline. The visceromotor response was assessed on the day before (day 0) and the day after (day 11) WAS or sham treatment (Fig. 1). EMG AUC was further normalized to response to 60-mmHg CRD on day 0 and expressed as a percentage of this baseline value. EMG signals were not recorded in 1 sham-treated rat due to equipment failure and not included in the visceromotor response analysis.

### Water avoidance stress protocol

The protocol was as described before [7]. Briefly, the test apparatus consisted of a plexiglas tank (45 cm length × 25 cm width × 20 cm height) with a block (8 cm length × 8 cm width × 10 cm height) affixed to the center of the floor of the tank. The tank was filled with fresh room temperature water (25°C) to within 1 cm of the the top of the block. The animals were placed on the block for a period of 1 hour daily for 10 consecutive days. Sham treatment consisted of placing the rats in the dry tank for 1 hour daily.

### Cerebral perfusion

On day 11, animals were allowed to rest for 15 min following the last distension of the CRD series. A piece of silastic tubing was filled with radiotracer [^14^C]-iodoantipyrine (125 µCi/kg in 300 µL of 0.9% saline, American Radiolabelled Chemicals, St. Louis, MO, USA). The radiotracer-filled tubing was then connected to the animal's cannula on one end, and to a syringe filled with euthanasia agent (pentobabital 50 mg/mL, 3 M potassium chloride) on the other. The animal was allowed to rest for another 5 min before receiving one episode of 60-mmHg. Thirty-five seconds after the onset of the distension, radiotracer was infused at 2.25 mL/min by a motorized pump, followed immediately by 0.7 mL of euthanasia solution, which resulted in cardiac arrest within ∼10 s, a precipitous fall of arterial blood pressure, termination of brain perfusion, and death [22]. This 10-s time window provided the temporal resolution during which the distribution of regional CBF (rCBF)-related tissue radioactivity was mapped. Half of each treatment group received no distension (0 mmHg) during CBF mapping and served as controls.

### Brain slicing and autoradiography

Brains were rapidly removed, flash frozen in methylbutane on dry ice (∼−55°C) and embedded in optimal cutting temperature compound (Sakura Finetek Inc., Torrance, CA,USA). Brains were subsequently sectioned on a cryostat (HM550 series, Microm International GmbH, Walldorf, Germany) at −18°C into 20-µm thick coronal slices, with an inter-slice spacing of 300 µm. Slices were heat-dried on glass slides and exposed to Kodak Biomax MR films (Eastman Kodak, Rochester, NY, USA) for 3 days at room temperature. Images of brain sections were then digitized on an 8-bit gray scale using a voltage stabilized light box (Northern Lights Illuminator, Interfocus Imaging Ltd., Cambridge, UK) and a Retiga 4000R charge-coupled device monochrome camera (Qimaging, Surrey, Canada). Autoradiographic CBF mapping in rodents has a spatial resolution of 100-µm, and hence, can provide information on sub-regional activation, such as in individual amygdaloid nuclei.

### Functional brain mapping data analysis

rCBF-related tissue radioactivity was quantified by autoradiography and analyzed on a whole-brain basis using statistical parametric mapping (SPM, version 5, Wellcome Centre for Neuroimaging, University College London, London, UK). Recently, we and others have developed and validated an adaptation of SPM for use in rodent brain autoradiograph [23]. In preparation for the SPM analysis, a 3-dimensional reconstruction of each animal's brain was conducted using 57 serial coronal sections (starting at ∼ bregma +4.5 mm) with a voxel size of 40 µm ×300 µm ×40 µm. Adjacent sections were aligned manually in Photoshop (version 9.0, Adobe Systems Inc., San Jose, CA, USA) and using TurboReg, an automated pixel-based registration algorithm implemented in ImageJ (version 1.35, http://rsbweb.nih.gov/ij/). This algorithm registered each section sequentially to the previous section using a nonwarping geometric model that included rotations and translations (rigid-body transformation) and nearest-neighbor interpolation. One “artifact free” brain was selected as reference. All brains were spatially normalized to the reference brain in SPM. Spatial normalization consisted of applying a 12-parameter affine transformation followed by a nonlinear spatial normalization using 3D discrete cosine transforms. All normalized brains were then averaged to create a final rat brain template. Each original 3D-reconstructed brain was then spatially normalized to the template. Normalized brains were smoothed with a Gaussian kernel (FWHM  = 3× voxel dimension in the coronal plane). A nonbiased, voxel-by-voxel analysis of regional brain activation was performed. Voxels for each brain failing to reach a specified threshold in optical density (70% of the mean voxel value) were masked out to eliminate the background and ventricular spaces without masking gray or white matter. We implemented a Student's *t*-test at each voxel. For each treatment (WAS or sham), a *t*-contrast was calculated comparing the 60-mmHg CRD to the 0-mmHg control subgroup. Threshold for significance was set at *P*<0.05 at the voxel level and an extent threshold of 100 contiguous voxels. This combination reflected a balanced approach to control both type I and type II errors. The minimum cluster criterion was applied to avoid basing our results on significance at a single or small number of suprathreshold voxels. Brain regions were identified according to a rat brain atlas [24]. In addition, we ran a factorial analysis to identify rCBF changes reflecting WAS x CRD interaction. Threshold for significance was set at *P*<0.05 at the voxel level and an extent threshold of 100 contiguous voxels. Data interpretation was focused on gray matter.

### Structurally linked functional connectivity of the prelimbic cortex

To test the hypothesis that WAS may result in altered corticolimbic modulation during noxious visceral stimulation, we applied seed-region correlation analysis to assess differences in the CRD-evoked FC of the PrL of the PFC between treatment groups. The seed region of interest (ROI) was hand drawn in MRIcro (version 1.40, http://cnl.web.arizona.edu/mricro.htm) for the right hemisphere over the template brain according the rat brain atlas [24] and intersected with clusters defining regional functional activation in the PrL area based on the SPM analysis. The result was one unilateral seed ROI representing the PrL region for each treatment type showing CRD-evoked functional activation. Mean optical density of the seed ROI was extracted for each animal using the MarsBaR toolbox for SPM (version 0.42, http://marsbar.sourceforge.net/). Correlation analysis was performed in SPM for each 60-mmHg CRD subgroup using the seed values as a covariate. Threshold for significance was set at *P*<0.05 at the voxel level and an extent threshold of 100 contiguous voxels. Regions showing significant correlations (positive or negative) in rCBF with the PrL are considered functionally connected with the PrL.

Anatomical (structural) connectivity of the PrL in the rat was extracted from BAMS. BAMS includes a large set of rat structural connections collated from the literature, or directly inserted by neuroanatomists [20], [21]. The collation methodology of neuroanatomical data employed in BAMS is fully described in Bota et al. [20]. Briefly, the connectivity patterns of gray matter regions are collated as reported in the published references recorded in BAMS, or as inferred by collators. The connectivity information is collated from the textual descriptions, and from the maps associated with references. Each connectivity report is associated with a qualitative strength, and with supporting textual annotations. Connectivity reports inferred by collators are associated with textual annotations that describe the annotation process. Overall, the rat PrL is associated with about 1600 connectivity reports in BAMS, with 106 gray matter regions that receive inputs from it and 177 regions that send outputs to it. The inputs of the rat PrL are associated with 37 references, and its outputs 62 references. The set of qualitative strengths of neuroanatomical connections collated in BAMS includes 10 values. Here, this set was encoded on a linear scale from 1 to 7, with 1 being very strong and 7 being very weak. Regions with ‘very weak’ connection to or from PrL, as well as connectivity reports with the strength ‘fibers of passage’, were removed for simplification. Only ipsilateral connections were included due to incomplete understanding of cross-hemispheric connectivity.

**Figure 2:**
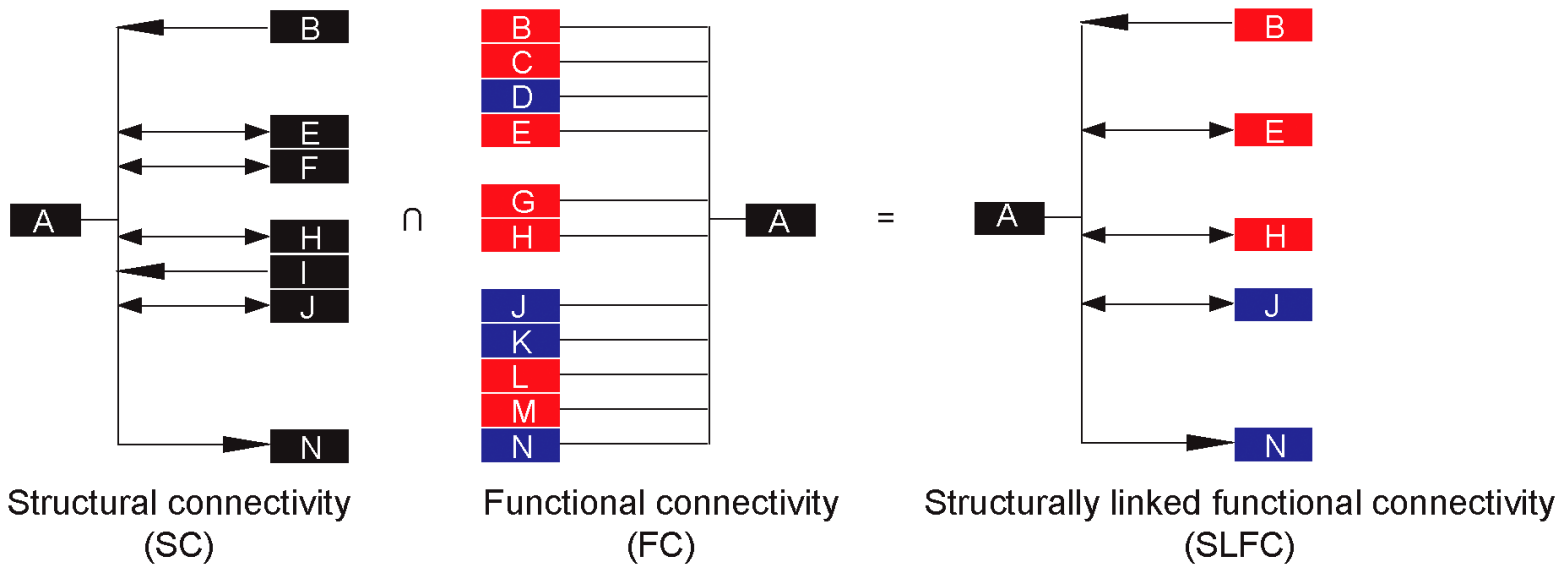
Structurally linked functional connectivity. Structural (SC) and functional connectivity (FC) networks are combined to create a structurally linked functional connectivity (SLFC) network such that final network contains all regions that the SC and FC networks have in common. Note the SLFC network inherits directionality information (denoted with arrows) from the SC network and the sign of functional interaction (positive/red or negative/blue correlation) from the FC network.

Results of the SPM seed correlation analysis were only analyzed for those regions structurally connected with the PrL. This is equivalent to taking an intersection of the structural and functional connectivity network of the PrL, resulting in an SLFC network (Fig. 2).

## Results

### Water avoidance stress induced visceral hyperalgesia

**Figure 3:**
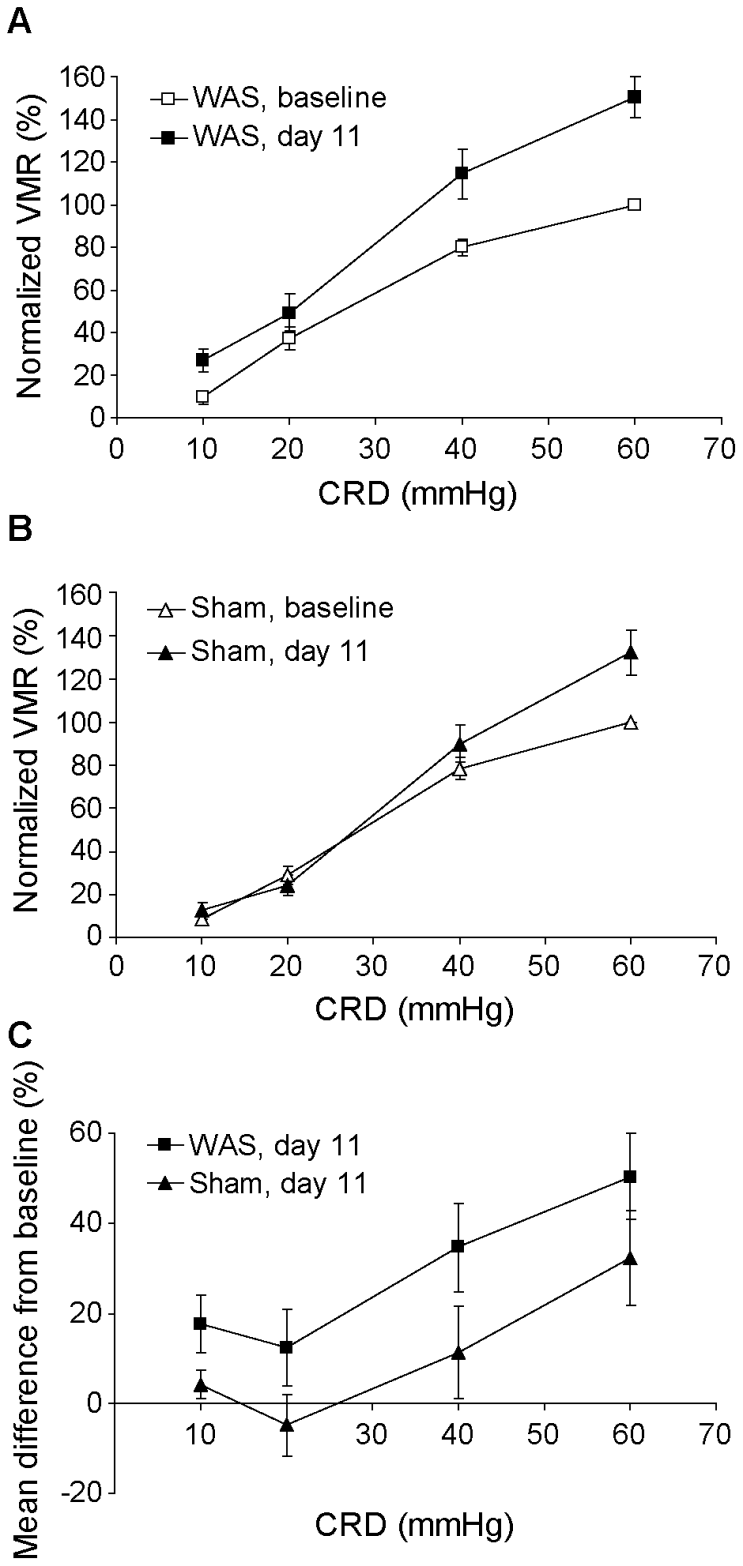
Effect of repeated water avoidance stress (WAS) on visceromotor response (VMR) to colorectal distension (CRD). (A) VMR measured as electromyographic (EMG) area under the curve (AUC) and expressed as % of control (baseline VMR to 60-mmHg CRD) was significantly increased following WAS on day 11 compared to day 0 baseline (*n* = 20, *F*(1, 19) = 18.19, *P*<0.001, two-way repeated measures ANOVA). (B) Repeated sham procedure caused a moderate increase in VMR, primarily to 60-mmHg CRD (*n* = 18, *F*(1,17) = 4.67, *P* = 0.045, two-way repeated measures ANOVA). (C) Compared to sham-treated rats, stressed rats showed significantly greater increases in VMR after treatment (*F*(1,36) = 4.47, *P* = 0.042, mixed model ANOVA). Data are expressed as means ± SEM.

Ten days of WAS induced significant increases in visceromotor response to CRD on day 11 as compared to day 0 baseline (Fig. 3A, *n* = 20, main effect of ‘Day’ *F*(1, 19) = 18.19, *P*<0.001, two-way repeated measures ANOVA with ‘Day’ and ‘CRD’ as the within-subjects factors). In comparison, the visceromotor response was only moderately increased in the sham-treated animals primarily due to an increase in response to 60-mmHg CRD (Fig. 3B, *n* = 18, main effect of ‘Day’, *F*(1,17) = 4.67, *P* = 0.045, two-way repeated measures ANOVA). Compared to the sham condition, stressed rats showed significantly greater increases in the visceromotor response on day 11 (Fig. 3C, main effect of ‘Treatment’, *F*(1,36) = 4.47, *P* = 0.042, mixed model ANOVA with CRD as the within-subjects factor and ‘Treatment’ the between-subjects factor).

### Comparison of CRD-evoked functional brain activation in WAS- and sham-treated rats

**Figure 4:**
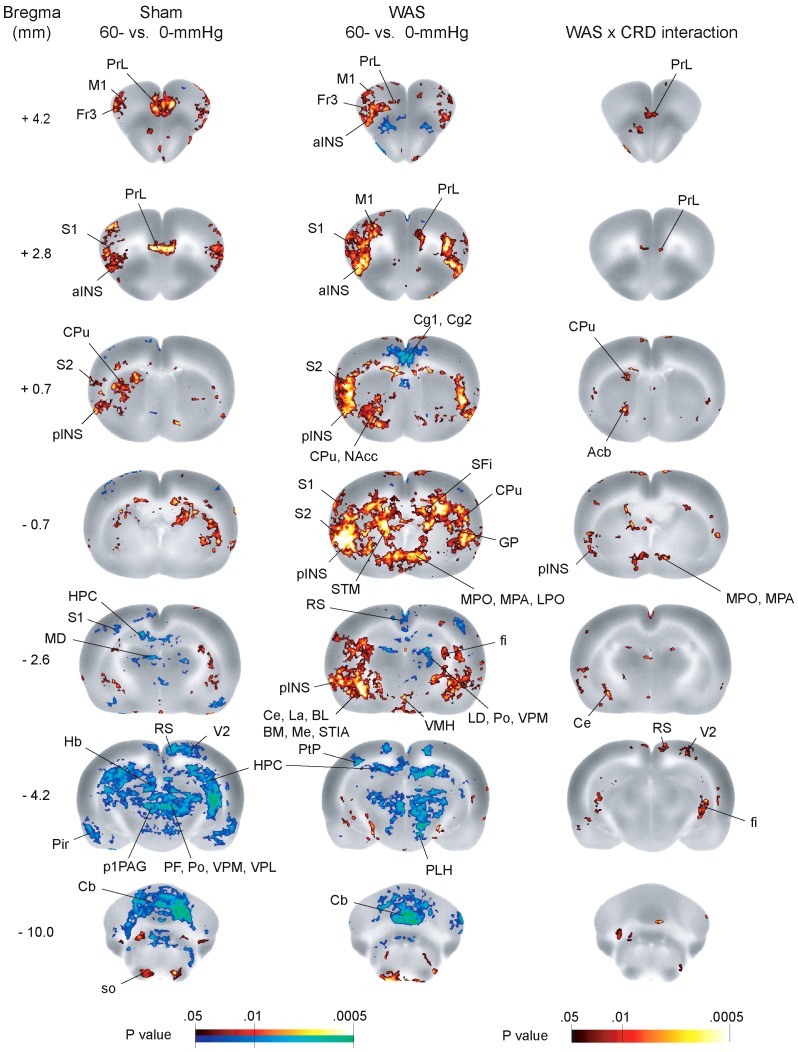
Comparison of colorectal distension (CRD)-induced functional brain activation in stressed and sham rats. Statistically significant increases (red scale) and decreases (blue scale) in regional cerebral blood flow (rCBF) contrasting the subgroup receiving 60-mmHg CRD and the one receiving no distension (0-mmHg) are shown for the sham (left column) and water avoidance stress (WAS)-treated rats (middle column) over representative coronal slices of the brain template with anterior-posterior coordinates given relative to the bregma (*n* = 9 or 10/subgroup). Results of factorial analysis are also colorcoded showing regions with significant WAS x CRD interaction (right column). Abbreviations: aINS (anterior insular cortex), BL (basolateral amygdaloid n.), BM (basomedial amygdaloid n.), Cb (cerebellar lobule), Ce (central amygdaloid n.), Cg1/Cg2 (cingulate ctx. area 1/area 2), CPu (caudate putamen), fi (fimbria), Fr3 (frontal ctx. area 3), GP (globus pallidus), Hb (habenular n.), HPC (hippocampus), La (lateral amygdaloid n.), LD (laterodorsal thalamic n.), LPO (lateral preoptic area), M1 (primary motor ctx.), MD (mediodorsal thalamic n.), Me (medial amygdaloid n.), MPA (medial preoptic area), MPO (medial preoptic n., medial part), NAcc (n. accumbens), RS (retrosplenial ctx.), p1PAG(p1 periaqueductal gray), PF (parafascicular thalamic n.), pINS (posterior insular ctx.), PLH (peduncular part of lateral hypothalamus), Pir (piriform ctx.), Po (posterior thalamic n.), PrL (prelimbic ctx.), S1 (primary somatosensory ctx.), S2 (secondary somatosensory ctx.), SFi (septofimbrial n.), SO (superior olive), STIA (bed nucleus of the stria terminalis, intraamygdaloid division), STM (bed nucleus of the stria terminalis, medial division), V2 (secondary visual ctx.), VMH (ventromedial hypothalamic n.), VPL/VPM (ventral posteromedial/posterolateral thalamic n.). The left side of each coronal image represents the left side of the rodent brain.

CRD-evoked functional brain activation was assessed for each treatment type by contrasting the subgroup receiving 60-mmHg CRD, and the one receiving no CRD (0-mmHg control) of the same treatment type (Fig. 4). Sham-treated rats showed CRD-evoked functional activation (increase in rCBF) in the PrL, primary motor, frontal area 3, primary and secondary somatosensory, anterior and posterior insular, and temporal association cortices, as well as in the dorsal caudate putamen, amygdala (lateral amygdaloid n., central amygdaloid n.), and superior olive. Sham rats also showed CRD-evoked deactivation (decrease in rCBF) in the retrosplenial, entorhinal, piriform, and secondary visual cortices, hippocampus, subiculum, thalamus (habenular n., mediodorsal n., posterior n. group, parafascicular n., ventral posteromedial n., ventral posterolateral n.), cerebellum (cerebellar lobule, cerebellar hemisphere), and areas of the brainstem (superior colliculus, dorsomedial periaqueductal gray, red n.; caudal linear n. of the raphe).

In contrast, WAS-treated rats showed CRD-evoked brain activation in a drastically different pattern. Major differences were noted in the magnitude and extent of regional activation. Greater activation in the WAS rats was noted in the anterior and posterior insula, and in the amygdala (central n., lateral n., basolateral n., basomedial n., medial n., bed nucleus of the stria terminalis intraamygdaloid division). The WAS rats also showed significant activation in the hypothalamus (medial preoptic area, medial preoptic n., lateral preoptic area, ventromedial hypothalamic n.), nucleus accumbens, and bed nucleus of the stria terminalis (medial division), which was not seen in the sham rats. Whereas sham rats showed activation in the anterior dorsal aspect of caudate putamen, WAS rats showed activation in the posterior and anterior ventral aspects of caudate putamen. Importantly, *reduced* activation in the PrL was noted in the WAS rats compared to sham. Deactivation in the cingulate cortex area 1 and 2 (Cg1, Cg2) was noted in the WAS but not the sham rats. WAS rats showed a similar pattern of deactivation as that seen in the sham condition, though to a lesser extent. Factorial analysis confirmed significant WAS x CRD interaction in the PrL, posterior insula, retrosplenial cortex, secondary visual cortex, anterior striatum, accumbens nucleus, bed nucleus of the stria terminalis medial division, medial preoptic nucleus, hippocampus, and central nucleus of the amygdala.

### Differences in the structurally linked functional connectivity of the prelimbic cortex

**Figure 5:**
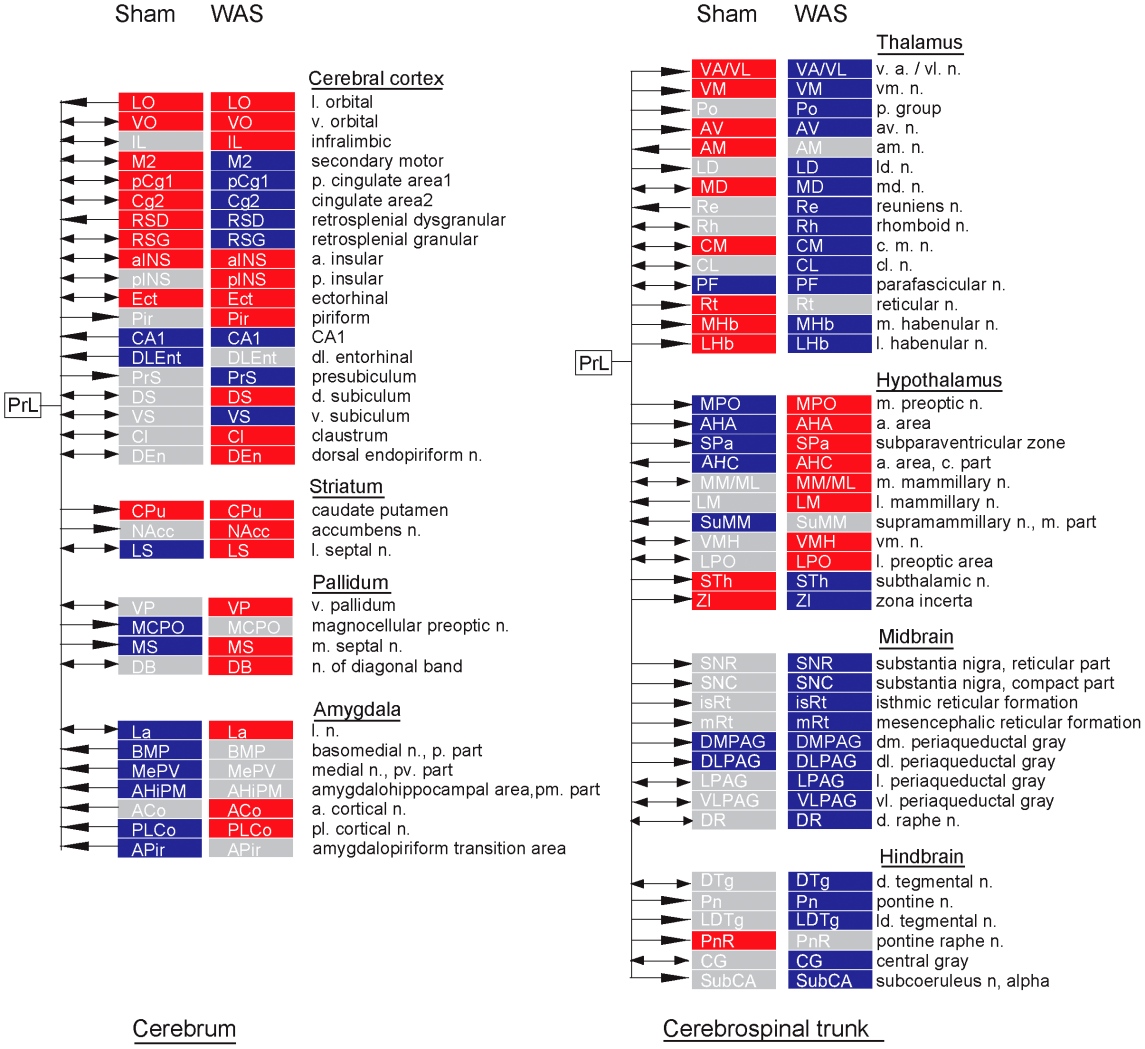
Stress-induced changes in the structurally linked functional connectivity (SLFC) of the prelimbic cortex (PrL) during colorectal distension (CRD). The SLFC network contains both directionality information of the underlying structural connections (denoted with arrows) and the sign of functional interaction (positive/red or negative/blue statistically significant correlation).

Seed correlation-based FC analysis was constrained by SC information of the PrL to generate SLFC of the PrL cortex (Fig. 5). In sham rats, PrL/PFC SLFC during noxious visceral stimulation was characterized by negative FC with the amygdala, whose nuclei provide either afferent input (basomedial n., posterior part; medial n., posteroventral part; amygdalohippocampal area, posteromedial part; posterolateral cortical n.; amygdalopiriform transition area) (PrL[-]←Amygdala) or bidirectional connections of the lateral amygaloid nucleus to the PrL (PrL[-]←→La). In addition, sham treated rats demonstrated the following SLFC of PrL cortex during noxious visceral stimulation:

1. Positive FC with a cluster of cortical regions over largely bidirectional structural connections, including orbital (ventral), secondary motor, cingulate, retrosplenial (dysgranular, granular), anterior insular, and ectorhinal cortices (PrL[+]←←Ctx).

2. Positive FC over projections to the striatum (PrL[+]←Striatum) or from lateral orbital cortex (PrL[+]←LO).

3. Negative FC over afferent projections from the hippocampal formation (CA1, dorsolateral entorhinal cortex) (PrL[-]←Hippocampal formation).

4. Negative FC with the hypothalamus over efferent projections to medial preoptic nucleus, the anterior hypothalamic area (anterior part), and subventricular zone (PrL[-]←Hypothalamus), and afferent projections from anterior hypothalamic (central part) and supramammillary nucleus (PrL[-]←Hypothalamus).

5. Positive FC with the thalamus over efferent projections to the ventral anterior, ventral lateral, anterior ventral, and reticular nuclei, and medial and lateral habenular nuclei (PrL[+]←Thalamus), as well as bidirectional connections with medial dorsal and central nuclei (PrL[+]←←Thalamus).

6. Negative FC over efferent projection to the dorsomedial and dorsolateral periaqueductal gray in the midbrain (PrL[-]←PAG).

WAS-induced changes in SLFC were most noticeable in relation to a cluster of cortical regions and to the amygdala. Whereas PrL in the sham animals showed significant, positive FC with a cortical cluster consisting of secondary motor, dorsal posterior cingulate (pCg1), ventral cingulate (Cg2), retrosplenial (dysgranular, granular) cortices, this connectivity turned *negative* in the stressed rats. In the sham rats, negative FC was noted between the PrL and the amygdala whose nuclei provide afferent input (basomedial n., posterior part; medial n., posterior ventral part; amygdalohippocampal area, posterior medial part; posterolateral cortical n., amygdalopiriform transition area) or bidirectional connections (lateral n.) to the PrL. In contrast, in the stressed rats, this negative functional connectivity was absent or turned positive (lateral n., anterior cortical n., posterolateral cortical n.).

In addition, WAS-induced alterations in SLFC of the PrL/PFC included the following:

1. Changes from positive to negative FC with the thalamus (ventral anterior, ventral lateral, anterior ventral, mediodorsal, centromedial, medial and lateral habenular nuclei) (PrL[-]→/←/← →Thalamus), subthalamic nucleus, and zona incerta.

2. Changes from negative to positive FC with the hypothalamus (medial preoptic n.; anterior hypothalamic area, anterior and central parts; subventricular zone) (PrL[+]→/←Hypothalamus).

3. New (no FC in sham rats) positive FC with infralimbic, posterior insular, and piriform cortices (PrL[+]→/← →Ctx), hypothalamus (medial mammillary n., ventromedial n., lateral preoptic area)(PrL[+]←/← →Hypothalamus), dorsal subiculum, nucleus accumbens, claustrum, dorsal endopiriform nucleus, ventral pallidum, and nucleus of the diagonal band.

4. New, negative FC with thalamus (posterior, reuinens, rhomboid, centrolateral nuclei)(PrL[-]→/←/← →Thalamus), and brainstem areas (substantia nigra reticulata and compacta, isthmic and mesencephalic reticular formation, lateral and ventrolateral periaqueductal gray, dorsal raphe n., dorsal and laterodorsal tegmental n., pontine n., central gray, subcoeruleus n. alpha)(PrL[-]→/← →Brainstem).

## Discussion

Neurobiological sequelae of chronic stress have been the subject of extensive research. For example, it has been well established that chronic stress in rodents can induce both visceral [6], [7], as well as somatic hypersensitivity [25]–[27]. Few studies have examined stress-induced changes in functional activation of brain nociceptive circuits, which can bring new insights into the underlying mechanisms. Using autoradiographic, perfusion-based functional brain mapping, we studied regional and network-level neural correlates of WAS-induced visceral hyperalgesia in awake, nonrestrained rats. Our main findings were that stressed rats showed greater CRD-evoked activation than sham-treated rats in the amygdala, insular cortex, and hypothalamus, but *reduced* activation in the prelimbic area of PFC (PrL/PFC). Profound differences between the stressed and sham rats were noted in the structurally linked functional connectivity of the PrL/PFC with cortical, limbic and brainstem areas. In particular, while negative functional connections were noted between the PrL/PFC and amygdala in the sham condition, these were absent in the stressed rats. These findings, in association with an exaggerated visceromotor response to CRD in the stressed animals, provide direct evidence that stress amplifies sensory and affective responses to nociceptive stimuli, with impairment of PFC-mediated pain modulation as a candidate central mechanism for stress-induced visceral hyperalgesia. We focus the discussion on stress-induced changes.

### Stress-induced changes in activation of amygdala and PFC evoked by noxious CRD

In the current study, stressed compared to sham treated rats showed greater CRD-evoked activation broadly across the amygdala, with the central amygdaloid nucleus showing the most significant differences. These findings are consistent with an extensive literature on stress induced sensitization of the amygdala. For example, the amygdala has been implicated in chronic stress-induced sensitization of anxiety- and fear-related responses to an acute stressor [28]–[30]. In particular, both chronic stress and chronic pain can increase excitability and responsiveness of subsets of neurons of the central n., a primary output of the amygdala to brain regions involved in autonomic regulation [31], [32]. Chronic restraint stress has also been shown to increase excitability of pyramidal neurons in the lateral amygdaloid nucleus [33], as well as to increase the number of dendritic spines in the amygdala [34]. Such stress-induced neural plasticity may mediate enhanced responses to noxious visceral stimulus. Further, chronic local application of the stress hormone corticosterone to the amygdala leads to visceral hyperalgesia, suggesting the amygdala as a site where stress hormone can modulate visceral nociception [35].

Activation of the PrL during acute visceral pain in nonstressed rats has previously been reported [10], [36]. In the current study, stressed compared to sham treated rats showed a striking hypoactivation in the PrL in response to CRD. The PrL is a part of the medial PFC in rodents. This brain region is thought to have features of dorsolateral PFC and anterior cingulate cortex of primates [37]–[41]. The PFC is a brain area vulnerable to chronic stress. For example, chronic restraint stress [42]–[44] and corticosterone administration [45] have both been shown to induce retraction of dendrites and loss of synaptic connections in the PFC, in line with reduced functional activation as observed in the current study. Chronic psychosocial stress in human subjects impairs PFC-dependent attentional control and disrupts FC within a frontoparietal network, including the PFC [46]. In contrast to results of the current study, Gibney et al. [36] reported exaggerated c-fos expression in the PrL in visceral hypersensitive Wistar-Kyoto rats compared to control Sprague-Dawley rats, albeit in the absence of a chronic stressor. These differences in PrL changes associated with visceral hyperalgesia may be attributable to different animal models of visceral hyperalgesia used, different experimental protocols (anesthetized vs. awake rats, no stress vs. WAS), and different modalities of measurement (c-fos vs. cerebral blood flow).

### Structurally linked functional connectivity analysis and connectome

Functional interaction between brain regions can be analyzed through FC analysis of brain imaging data. Here, we focused FC analysis on the PrL and constrained FC results with SC information of the PrL. The integration of whole brain-level FC and SC information reflects a recent trend in human brain imaging field to take advantage of rapid advances in the Human Connectome Project [47]. With the Mouse Connectome Project (http://www.mouseconnectome.org/), BAMS [20], and other rodent structural connectome projects well underway, a similar approach has become feasible for animal research. Based on the complete information of the SC of the PrL collated in BAMS, we were able to thoroughly investigate stress-induced changes in SLFC of the PrL. The first advantage of SLFC over regular FC is the simplification of the connectivity network by the removal of FC connections not substantiated by structural projections. FC connections without SC may be false positive findings, or indirect FC connections. The second advantage of SLFC is that it combines SC directionality with the sign of FC. The resulting SLFC can help generate new hypotheses about the causality in the brain circuits. While both SC and FC data were binarized for simplification in this study, the strength of each SC and FC connection can also be integrated into SLFC analysis. The current study represents one of the first rodent studies to present a detailed integration of structural information into FC analyses.

### Structurally linked functional connectivity of the PFC-amygdala circuit and the effects of stress

In rodents, the PrL is reciprocally connected with the lateral and basolateral nuclei of the amgydala, and sends efferent projections to the central nucleus and anterior part of basomedial nucleus, and receives afferent projections from medial, posterior, and cortical nuclei and posterior part of the basomedial nucleus of the amygdala. In sham rats, PrL/PFC SLFC during CRD was characterized in our study by negative FC with the amygdala nuclei over mostly afferent projections from the amygdala (PrL[-]←Amygdala), except bidirectional connection with the lateral nucleus (PrL[-]← →La). These SLFC results suggest that in sham animals, PrL/PFC inhibits the amygdala through its projection to the lateral amygdalar nucleus, whereas the amygdala may in return inhibit PrL/PFC activity though its lateral, basomedial, medial, and cortical nuclei.

Bidirectional PFC-amygdala interactions have been extensively studied in humans and in laboratory animals, and changes in these interactions have been implicated in the regulation of negative emotion, mood and pain [48]–[56]. The inhibitory interaction between the PrL/PFC and amygdala is likely bidirectional. On the one hand, it has been well documented that PFC regulates amygdala-mediated responses to aversive stimulus [57]. On the other hand, ample evidence exists for amygdala-mediated modulation of PFC [58], particularly under aversive condition [59], [60]. We have recently applied correlation-based FC and graph theoretical analysis to characterize brain activation at the network level in expectation of visceral pain [13]. Animals previously trained with a step-down passive avoidance paradigm using noxious CRD as the aversive stimulus demonstrated negative FC between the amygdala and areas of the PFC (including PrL and dorsal and ventral cingulate cortex, Cg1, Cg2) when reexposed to the conditioned context—a finding that was interpreted as evidence for inhibitory corticolimbic modulation.

In the current study, stressed rats compared to sham rats showed substantial changes in the SLFC of PrL/PFC. The negative FC with the amygdala seen in the sham disappeared, and in its place appeared a few positive connections (with the lateral and corticoamygdaloid nuclei). Our results are consistent with those of Correll et al. [32] who using in-vivo extracellular neural recordings reported that chronic cold stress enhanced the acute footshock-induced response of the central amygdaloid nucleus, and that chronic stress weakened prefrontal inhibitory regulation of this response. Our data suggest similar chronic stress-induced disinhibition of the amygdala by the PFC.

### Stress-induced changes in activation of other brain regions during noxious CRD

Previous imaging studies in animal and human subjects [reviewed by 61] have also implicated the anterior cingulate cortex in visceral pain processing and regulation, with the majority of visceral distension studies reporting enhanced activation of mid-cingulate subregions. Here, the observed deactivation in the cingulate area in the stressed rats and no activation in the sham rats was unexpected. Previously, we have reported cingulate activation in male, naïve rats (with no prior experience of CRD) receiving acute, noxious CRD [10]. In the current study, rats received a series of CRD eleven days prior, as well as 20 minutes prior to the CBF perfusion procedure. This difference in protocol may have contributed to this different pattern of cingulate activation. For example, CBF level in the cingulate areas in the 0-mmHg control rats may have been elevated from baseline due to prior exposure to repeated, noxious CRD. The exact cause and implication of cingulate deactivation in the stressed rats remains to be further investigated.

Whereas both sham and stressed rats showed CRD-evoked activation in the anterior and posterior insula, activation in the stressed rats was much greater in amplitude and extent. We have reported CRD-evoked activation of the anterior and posterior insula in normal (nonstressed), male rats [10], as well as activation of anterior insula in expectation of CRD [13]. Activation of the insula in response to acute rectal distension is the most consistently reported finding in human brain imaging studies [61], and alterations of insular functional activation have been reported in IBS patients. The posterior insula in its role as primary interoceptive cortex mediates sensory processing of pain and is part of a sensorimotor network, whereas the anterior insula is involved in a salience network closely linked to emotional arousal [62]. In the rat, anterior insular cortex may modulate pain processing through its projection to the amygdala and periaqueductal gray in the rat [63], [64].

Stressed rats also showed significant CRD-evoked activation in the hypothalamus, bed nucleus of stria terminalis, accumbens nucleus and ventral striatum, but deactivation in the cingulate cortex, which were all absent in the sham rats. The hypothalamus is believed to be modulated by PFC, and in turn regulates activity of descending inhibitory and facilitatory pathways through periaqueductal gray and pontomedullary nuclei [16], [65]–[67]. The ventromedial hypothalamus has also been implicated in the generation of the affective dimension of pain [68]. Increased hypothalamic activation to rectal distension has been observed in IBS patients as compared to healthy controls [69]. The bed nucleus of the stria terminalis, considered extended amygdala, receives heavy projection from the basolateral amygdala and projects to the hypothalamus and brainstem areas, and participates in anxiety and stress responses [70]. Chronic immobilization stress has been shown to induce dendritic remodeling of neurons of the bed nucleus of the stria terminalis [71]. Collectively, augmented activation of the hypothalamus, bed nucleus of the stria terminalis, and amygdala may underlie increased pain responses in the affective dimension, as well as increased descending facilitatory pain modulation.

The nucleus accumbens and ventral striatum participate in reward responses and positive emotional states. The accumbens nucleus and ventral striatum are also considered part of the emotional motor system [72], serving perhaps as a gateway between the limbic system and the motor system [73]. Human brain imaging studies have reported activation of these structures by noxious thermal stimuli [74], as well as in expectation of aversive somatic stimuli [75]. Interestingly, in a treatment study of IBS patients, Berman et al. [76] showed that Alosetron, a serotonin receptor antagonist, elicited decreases in rCBF in the amygdala, ventral striatum, and dorsal pons, in significant correlation with symptom (abdominal pain) reduction. Their findings suggest a possible role of the ventral striatum in central pain sensitization in IBS.

### Structurally linked functional connectivity of the PFC to other brain regions and the effects of chronic stress

During visceral noxious stimulation, sham treated rats demonstrated a positive SLFC of the PrL cortex with the thalamus, anterior insula and other cortical areas. This is consistent with an integrative role of PrL/PFC in the processing of visceral input. In addition, the negative connectivity of PrL/PFC with limbic areas (amygdala, hypothalamus) and periaqueductal gray in the midbrain is consistent with PFC-limbic-periaqueductal gray inhibitory modulation [77], [78]. Water avoidance stress induced substantial changes in the SLFC of the PrL with the thalamus, hypothalamus, and brainstem. Connections with the thalamus which demonstrated significant positive FC in the sham, appeared significantly negative in the WAS rats, whereas FC showed the reverse pattern with the hypothalamus (excluding the subthalamic n., and zona incerta). In the stressed rats, but not the sham, PrL demonstrated significant negative FC with areas of the brainstem, including the substantia nigra, reticular formation, periaqueductal gray, dorsal raphe nucleus, tegmental nucleus, pontine nucleus, central gray, and subcoeruleus nucleus alpha. This complex pattern of alterations in SLFC suggests profound changes in how the PrL/PFC interacts with other cortical and subcortical areas during visceral pain processing as a result of chronic stress.

### Translational implications of rodent brain imaging findings in the study of pain

Human functional brain imaging has been extensively applied to investigate central processing and modulation of pain, including visceral pain, and to characterize alterations in central pain responses in functional pain disorders, including IBS [16], [61]. A recent quantitative meta-analysis of imaging data from 19 published studies reported that compared to healthy controls, IBS patients have greater engagement of regions associated with emotional arousal (perigenual anterior cingulate cortex and amygdala) and homeostatic afferent processing (anterior mid-cingulate cortex, medial thalamic regions, mid-insula, areas of the midbrain). In contrast, controls show greater reliable activation in cortical regions involved in modulation of pain and emotion (lateral and medial prefrontal cortex, Brodmann Area 49), which is largely absent in IBS patients [79]. In striking agreement with these human brain imaging findings, the current study showed that stressed rats, compared to sham, had much greater activation in the insula and amygdala, but reduced activation in PrL, a PFC region with features of the dorsolateral PFC [37], [38] and the anterior cingulate cortex [40], [41] of primates. This adds to our previous reports of remarkable homology in functional brain activation between the rat and human in response to acute noxious CRD in both males and females, and in expectation of CRD in males [10], [12], [13].

In conclusion, chronic stress induced marked alterations in CRD-evoked functional brain activation characterized by *hypoactivation* of the prelimbic area of PFC and *hyperactivation* of the insular cortex, amygdala, and hypothalamus. Structurally linked functional connectivity analysis further revealed stress-induced disruption of PFC-limbic (amygdala and hypothalamus) inhibitory interaction during CRD. Dysfunction of the PFC, including impairment of the PFC-limbic regulatory circuit, is strongly implicated as a central mechanism contributing to stress-induced visceral hyperalgesia. The findings of hypoactivation in PFC and hyperactivation in limbic/paralimbic structures in the homeostatic afferent processing network and emotional arousal network are in general agreement with human brain imaging findings on altered brain responses to noxious visceral stimulus in IBS patients. These findings provide further support for the face and construct validity of the WAS animal model for human IBS. Functional brain mapping in awake, nonrestrained rodents can be a powerful tool for bridging animal and human visceral pain research, for gaining new mechanistic insights, and for preclinical drug evaluation with presumably greater predictive power [80]. Future work will need to evaluate if activation/deactivation patterns reported in our study would be different and allow for animal-to-human translation if another painful stressor were used (e.g. electric shock; noxious heat).
